# Treatment of Therapy-Refractory, Symptomatic Vasospasm in a Radial Artery Bypass Graft With Balloon Angioplasty in a Patient With Severe Subarachnoid Hemorrhage: A Case Study

**DOI:** 10.1227/neuprac.0000000000000158

**Published:** 2025-08-25

**Authors:** Franziska Meinert, Patrick Dömer, Levent Tanrikulu, Simeon O. A. Helgers, Claudia Klüner, Johannes Woitzik, Christian Mathys

**Affiliations:** * Department of Neurosurgery, Carl von Ossietzky University Oldenburg, Oldenburg, Germany;; ‡ Research Center Neurosensory Science, Carl von Ossietzky University Oldenburg, Oldenburg, Germany;; § Institute of Radiology and Neuroradiology, Evangelisches Krankenhaus Oldenburg, Carl von Ossietzky University Oldenburg, Oldenburg, Germany;; ‖ Department of Diagnostic and Interventional Radiology, University Düsseldorf, Düsseldorf, Germany

**Keywords:** Balloon PTA (percutaneous transluminal angioplasty), Case report, Cerebral revascularization, Endovascular intervention, Graft vasospasm, High-flow bypass, Radial artery graft, Subarachnoid hemorrhage (SAH)

## Abstract

**BACKGROUND AND IMPORTANCE:**

This case highlights the effectiveness of endovascular balloon dilatation (percutaneous transluminal angioplasty (PTA)) for treating vasospasm in a radial artery (RA) bypass graft from the internal carotid artery to the M2 branch of the middle cerebral artery in a patient with severe subarachnoid hemorrhage.

**CLINICAL PRESENTATION:**

A 69-year-old woman with severe subarachnoid hemorrhage due to a ruptured giant aneurysm in the ophthalmic segment of the right internal carotid artery underwent an extracranial-intracranial high-flow bypass with a RA graft, followed by trapping of the aneurysm. During her intensive care unit stay, vasospasms developed primarily in the intracranial radial graft and M2 branches. These spasms showed only minimal improvement after standard care and intra-arterial vasospasmolysis with nimodipine. Thus, after systemic anticoagulation, balloon PTA was performed, treating both the intracranial and extracranial sections of the graft, including areas near the anastomosis. The procedure was complication-free, with immediate morphological success and significant improvement in perfusion to the middle cerebral artery territory.

**CONCLUSION:**

Endovascular balloon PTA should be considered a rescue measure for vasospasm in extracranial-intracranial bypasses, particularly with RA grafts post-SAH. Owing to its muscular structure, the RA graft is especially prone to spasm, potentially compromising graft patency. When pharmacological treatments fail, balloon PTA offers a targeted intervention to restore vessel caliber, stabilizing blood flow and preventing ischemic complications, thus supporting the bypass's long-term success.

**ABBREVIATIONS:**
EC-IC: extracranial-intracranial
RA: radial artery.

Cerebral revascularization through bypass surgery is a treatment strategy for complicated intracranial aneurysms that are not amenable to conventional clipping or endovascular techniques.^1-5^ Commonly, the great saphenous vein serves as a high-flow conduit but is limited by diameter mismatches and susceptibility to kinking.^1,6^ A good alternative is the establishment of an extracranial-intracranial (EC-IC) bypass using the radial artery (RA) as a conduit. However, as known from coronary bypass surgery, there is an increased risk of vasospasm, particularly at the anastomosis sites, which is associated with high risk of cerebral ischemia. Therefore, various groups have focused on the prophylaxis and treatment of vasospasm.^7-9^ For patients with refractory vasospasm, endovascular treatments, including topical application of calcium channel blockers or balloon angioplasty with or without stenting, are well-established options.^10,11^ The pressure distention technique described by Sekhar et al has been highlighted, alongside surgical and endovascular measures.^12-16^

Although endovascular intervention is established for coronary bypass, data on endovascular therapy for cerebral bypass grafts remain sparse. Ramanathan et al^13^ observed percutaneous transluminal angioplasty (PTA) as safe and effective for early perioperative vasospasm, highlighting PTA with stenting as a beneficial long-term strategy.

Vasospasm is common in subarachnoid hemorrhage (SAH), driven by a biochemical cascade from blood release into the subarachnoid space.^17,18^ In this case, SAH-related vasospasm affected the anterior and middle cerebral arteries, and the distal extracranial and the intracranial segments of an EC-IC RA bypass graft.

Case reports document PTA for managing perioperative vasospasm. In our case, this approach was successfully applied to severe SAH-induced vasospasm within a RA bypass graft after a ruptured giant aneurysm. This case report follows the Surgical CAse REport (SCARE) Criteria.^19^

## CLINICAL PRESENTATION

**FIGURE 1.**
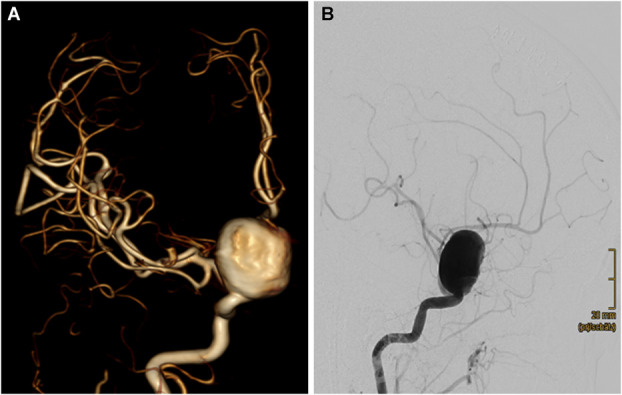
**A**, 3-dimensional angiographic representation of the aneurysm on the ophthalmic segment of the right internal carotid artery, measuring 2.2 × 2.2 × 1.6 cm in coronal view; **B**, Digital subtraction angiography in sagittal view.

A 69-year-old female patient presented with SAH, classified as World Federation of Neurosurgical Societies (WFNS) grade II, Hunt and Hess grade III, and modified Fisher grade IV. The hemorrhage originated from a ruptured aneurysm measuring 26 × 24 × 21 mm, located on the ophthalmic segment of the right internal carotid artery. These measurements represent the overall aneurysm dimensions, including a peripheral thrombotic component (see Figure 1A and 1B). Owing to the broad-based configuration of the aneurysm, stent-assisted coiling would have been required for endovascular treatment. An endovascular attempt was made; however, it was unsuccessful because of the very narrow curvature of the parent vessel, which prevented adequate stent opening and deployment of a braided stent. In addition, the maximum diameter of the available open-cell laser-cut stents was too small.

After microsurgical clipping was also deemed unsuitable, the interdisciplinary team opted for a high-flow EC-IC bypass using an RA graft, with subsequent vessel occlusion. The bypass, from the external carotid artery to an M2 branch, was performed successfully under temporary clipping. Bypass patency was assessed intraoperatively using indocyanine green videoangiography^20^ and laser speckle imaging. Laser speckle imaging showed satisfactory blood flow from baseline during carotid clipping, justifying intracranial trapping of the aneurysm. Postoperative angiography and computed tomography (CT) perfusion confirmed good bypass patency with satisfactory cerebral perfusion. Following SAH baseline therapy per German Society of Neurology (DGN) and German Society of Neurosurgery (DGNC) guidelines, the patient exhibited significantly delayed awakening.

CT angiography confirmed bypass patency; however, the patient developed vasospasm on day 3, affecting both the RA graft and M2 segments. Multiple rounds of endovascular vasospasmolysis with intra-arterial nimodipine were performed. On day 6, the external ventricular drain was replaced with a lumbar drain, averaging 5 mL/hour, totaling approximately 120 mL daily. As vasospasm in the RA graft worsened, limiting perfusion in the middle cerebral territory, a decision was made to proceed with balloon-assisted PTA.

**FIGURE 2.**
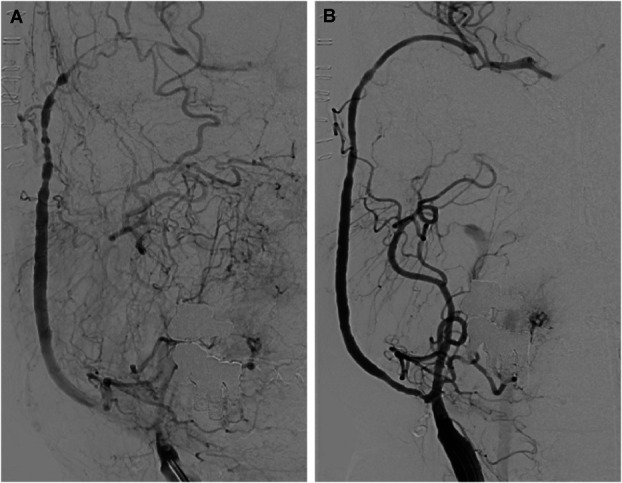
Digital subtraction angiography in coronal imaging. **A**, Radial artery bypass with stenosis due to vasospasm, primarily in the intracranial segment, along with vasospasm in the middle cerebral artery. **B**, Postinterventional assessment in the early arterial phase.

The patient received 5000 units of heparin intravenously. The catheter sheath was exchanged for an 8F introducer sheath, through which an 8F guiding catheter (Guider Softip XF, Boston Scientific) was advanced into the right common carotid artery. The bypass was probed using a 2.5 × 8 mm NeuroSpeed PTA balloon catheter (Acandis) over a microguidewire (Synchro SELECT, Stryker). Balloon PTA was then performed with 6 bar along multiple stenotic sections in both intracranial and extracranial portion of the bypass, including the proximal section near the anastomosis, achieving favorable morphological results. After risk-benefit assessment, slight stenosis near the M2 junction was left untreated. Additional stenting was not deemed necessary, allowing to continue with mono-antiaggregation with acetylsalicylic acid 100 mg/d for 6 weeks (see Figure 2).

**FIGURE 3.**
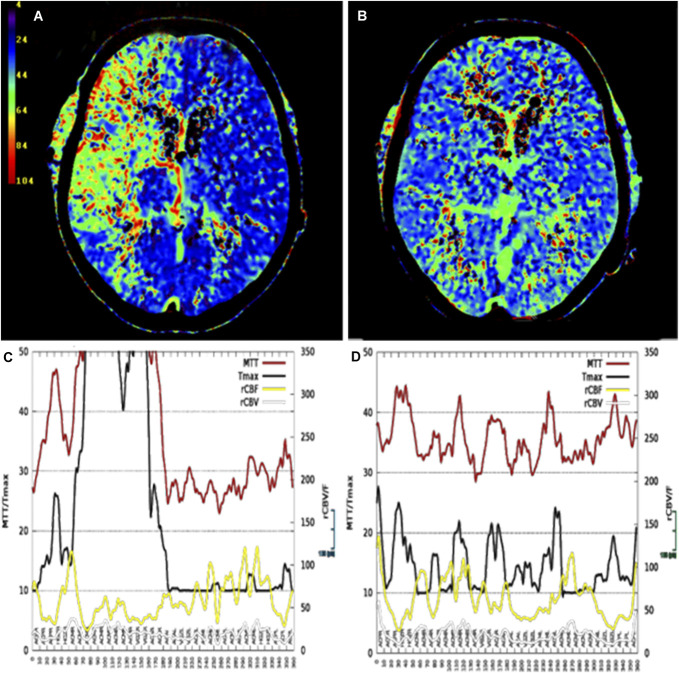
**A**, The color-coded map provides a visual representation of the MTT in tenths of a second [s*10^−1^ ] before dilatation, showing MTT prolongation in the anterior and middle cerebral artery territory. **B**, MTT map after dilatation with significant reduced MTT. **C**, This map shows a cortical ribbon analysis, which is generated by dividing the brain into 180 2°-segments, each averaged over 10°, covering a full 360° circle starting in the right posterior circulation territory and ending in the left posterior circulation territory. The 4 standard perfusion parameters are displayed, illustrating the increase in MTT and Tmax as well as the rCBF level in the critical segment with **D** normalization observed after dilation (This analysis was produced using Angiotux CT 2D, ECCET, Beck A., Aurich V.; www.eccet.de.). CBF, cerebral blood flow; MTT, mean transit time; rCBF, reduced CBF.

Comparison of preinterventional and postinterventional CT perfusion studies demonstrated a marked reduction of regional mean transit time prolongation from 5 to 3.5 seconds on the bypass side (right hemisphere).^21^ No new infarcts were detected (See Figure 3). No further flow-relevant vasospasms occurred. After prolonged intensive care unit treatment and early neurorehabilitation, the patient showed left hemiparesis with fine motor impairment of the hand, moderate proximal paresis in the left limbs, and persistent left hemineglect. With physiotherapy or a rollator, she was able to stand and walk.

### Informed Consent to Procedure

The procedure was performed with written informed consent obtained from the patient's legally authorized representative (her spouse), in accordance with institutional standards.

### Ethical Approval Statement

According to our institutional guidelines, ethical approval was not required for anonymized single-patient case reports with obtained consent for publication.

### Informed Consent to Publish

Written consent for publication was provided by the patient's legally authorized representative (husband) because of the patient's inability to consent. After full disclosure regarding publication and confidentiality, permission was granted for anonymized release of relevant clinical details. Consent covered the use of anonymized data for scientific publication in line with ethical standards.

### Disclosure of Off-Label Use

The CE-marked (no U.S. Food and Drug Administration–approval at time of publication) indicated use of the NeuroSpeed PTA balloon catheter (Acandis, Pforzheim, Germany) is the treatment of vasosclerotic cerebral stenosis. Therefore, the described application of the device has to be considered as off-label use.

## DISCUSSION

Endovascular interventions such as PTA, with or without stenting, are well-established procedures for coronary artery stenosis.^22,23^ PTA is also routinely used as a rescue measure after the exhaustion of conservative therapies, such as intravenous administration of nimodipine or intrathecal application of nicardipine, during the vasospasm phase in cases of subarachnoid.^10,11,24-26^ However, its use for vasospasm in RA bypass grafts has been approached with caution. To date, only a few cases have been documented, though these have shown highly favorable outcomes, including significant vasospasm reduction, improved flow through the bypass, and, consequently, enhanced cerebral perfusion.^14^ In this case, no complications were observed directly related to PTA. Immediate and very good morphological results were achieved, with a significant improvement in perfusion in the middle cerebral artery territory.

However, invasive procedures should be approached with caution. Potential risks include dissection and/or occlusion of the conduit, which could lead to hypoperfusion and infarction, as well as fatal SAH because of rupture of the bypass vessel or its intracranial anastomosis. Larger case series and extended follow-up periods are needed to establish a reliable risk-benefit profile, enabling more precise conclusions about the efficacy and safety of this intervention.

## CONCLUSION

Endovascular balloon dilatation should be regarded as a viable rescue strategy for vasospasm occurring after the creation of an EC-IC bypass in a patient with SAH, particularly when the RA is used as the graft conduit. This technique might be especially valuable for treating graft vasospasm, providing a critical therapeutic option when other conservative measures fail.

## COMMENTS

The authors present a difficult case of a large aneurysm treated with parent vessel occlusion and radial artery bypass which developed severe angiographic vasospasm in both the graft and the intracranial vasculature. They report successful treatment with angioplasty after intra-arterial pharmacotherapy was not effective. The angiographic and physiologic improvement the authors achieved is impressive. The authors fortunately didn't need to angioplasty directly at the distal anastomosis site due to the appropriate wide “fish mouth” of the distal anastomosis, which is an important technical nuance. The mechanism of vasospasm in this case is not the same mechanism as in intracranial vasculature, which is more likely related to subarachnoid blood products and related inflammation, spreading depolarization, and other factors. This may be relevant in that even interventions that may improve delayed cerebral ischemia such as early lumbar drainage would not be expected to affect this early graft vasospasm. The problem of radial artery graft vasospasm was described in the early cases of Sekhar^1a^; however, with the introduction of the pressure distension technique, it was essentially completely mitigated in later cases. It is an important nuance that this pressure distention procedure must be done sequentially in small segments along the length of the vessel to “pop” the vessel open. The authors have reported an important bail-out strategy with good angiographic results in case this pressure distention fails or was not performed aggressively enough.

**Andrew P. Carlson**

*Charlottesville, Virginia, USA*
